# The diabetes gene *Tcf7l2* organizes gene expression in the liver and regulates amino acid metabolism

**DOI:** 10.1016/j.molmet.2025.102208

**Published:** 2025-07-15

**Authors:** Joanna Krawczyk, William O’Connor, Pedro Vendramini, Mareike Schell, Kiran J. Biddinger, Matt Kanke, George Pengo, Ivana Semova, Tiffany Fougeray, Marcia Haigis, Krishna G. Aragam, Wouter H. Lamers, Linus T. Tsai, Praveen Sethupathy, Sudha B. Biddinger

**Affiliations:** 1 Division of Endocrinology, Boston Children’s Hospital, Harvard Medical School, Boston, MA, USA; 2 Cardiovascular Disease Initiative, Broad Institute of MIT and Harvard, Cambridge, MA, USA; 3 Cardiovascular Research Center, Massachusetts General Hospital, Harvard Medical School, Boston, MA, USA; 4 Center for Genomic Medicine, Department of Medicine, Massachusetts General Hospital, Harvard Medical School, Boston, MA, USA; 5 Department of Biomedical Sciences, Cornell University, Ithaca, NY, USA; 6 Department of Cell Biology, Blavatnik Institute, Harvard Medical School, Boston, MA, USA; 7 Tytgat Institute for Liver and Intestinal Research, Academic Medical Center, University of Amsterdam, Meibergdreef 69-71, 1105 BK, Amsterdam, the Netherlands; 8 Division of Endocrinology, Diabetes and Metabolism, Beth Israel Deaconess Medical Center, Boston, MA, USA; 9 Broad Institute of Harvard and MIT, Cambridge, MA, USA; 10 Department of Molecular Metabolism, Harvard T.H. Chan School of Public Health, Boston, MA, USA

**Keywords:** Zonation, Transcription, Diabetes, Metabolism

## Abstract

**Objective:**

Though *Tcf7l2* harbors the strongest genetic association with diabetes identified thus far, how it promotes metabolic disease remains unclear. Our aim was to dissect the contribution of hepatic TCF7L2.

**Methods:**

Mice with liver-specific knockout of *Tcf7l2* produced by targeted deletion of exon 1 were subjected to physiological characterization, single nucleus sequencing, and metabolite profiling. In parallel, a phenome-wide association study was performed in humans.

**Results:**

We found that liver-specific deletion of *Tcf7l2* had little effect on plasma glucose, but disrupted hepatic zonation. That is, many genes normally show gradients of expression across the liver lobule; in the absence of *Tcf7l2*, these gradients collapsed. One major consequence was the disorganization of glutamine metabolism, with a loss of the glutamine production program, ectopic expression of the glutamine consumption program, and a decrease in glutamine levels. In parallel, glutamine was found to be the most significantly decreased metabolite in the plasma of individuals harboring the rs7903146 variant in *Tcf7l2*.

**Conclusions:**

Taken together, these data indicate that hepatic TCF7L2 has a secondary role in glycemic control, but a primary role in maintaining transcriptional architecture and glutamine homeostasis.

## Highlights

• The diabetes gene *Tcf7l2* is crucial for maintaining hepatic zonation.
• Hepatocyte *Tcf7l2* deletion has minor effects on glucose homeostasis.
• TCF7L2 plays a central role in regulating glutamine metabolism.

## 1 Introduction

Genome-wide association studies are performed with the expectation that they will identify genes that are important in human physiology and disease. For type 2 diabetes, the major outcome of these efforts is the identification of *TCF7L2* [1]. TCF7L2 encodes a member of the TCF/LEF family of transcription factors, which bind to β-catenin – a transcriptional regulator that is responsive to Wnt signals [1,2]. In addition to being the first gene found to be associated with type 2 diabetes, *TCF7L2* remains the strongest and most significant association, replicated in multiple cohorts of different ancestries [3]. It appears to be involved in almost 20% of type 2 diabetes cases [4]. Furthermore, *TCF7L2* has been implicated in numerous other diseases associated with type 2 diabetes, ranging from cardiovascular disease to cancer to liver disease [5]. Yet, more than a decade after the identification of TCF7L2 as a diabetes gene, the functions of TCF7L2 remain unclear.

Whole body knockouts of *Tcf7l2* are hypoglycemic and die shortly after birth [6]. Knockout of *Tcf7l2* in β-cells promotes their dysfunction [7] whereas knockout in adipocytes leads to adipocyte hypertrophy, obesity and insulin resistance [8]. These studies suggest that TCF7L2 may act in multiple tissues to promote diabetes.

The effects of *Tcf7l2* disruption in the liver have been discordant, with some studies reporting hypoglycemia and others hyperglycemia [[9], [10], [11], [12], [13], [14]]. Similarly, both increased and decreased susceptibility to steatosis have been reported [[15], [16], [17]]. These discrepancies may be due, at least in part, to the complexities of dissecting TCF7L2 function. TCF7L2 is a member of the TCF/LEF family of transcription factors, which are often redundant with one another; moreover, both TCF7L2 and β-catenin have multiple additional binding partners [18,19]. Some mutations used to disrupt TCF7L2 function lacked the β-catenin interaction domain, but retained the DNA binding domain [14]; others lacked the DNA binding domain but retained the β-catenin interaction domain [12]. Though both mutations disrupt TCF7L2, their secondary effects on β-catenin and the other members of the TCF/LEF family could differ, making it difficult to define the unique functions of TCF7L2.

Here, we use mice with targeted deletion of exon 1, with a complete loss of function [20], to define the specific effects of TCF7L2 in the liver. We find that hepatic TCF7L2 has little effect on glucose and lipid metabolism; however, it is essential for the organization of gene expression in the liver. *Tcf7l2* deletion leads to diverse metabolic effects, particularly a decrease in glutamine, which is also observed in individuals with the single-nucleotide polymorphism (SNP) rs7903146 in *TCF7L2*.

## 2 Materials and methods

### 2.1 Animals

*Tcf7l2*^*Flox/Flox*^ mice [20] were purchased from Jackson Laboratories (strain code: 031436) and group-housed at 21 °C on a 12 h light/dark cycle (7 am/7 pm) with free access to food, unless otherwise indicated. At the end of the experiment, mice were killed in the *ad libitum* state at 2 pm. All animal experiments were performed under approval of the Institutional Animal Care and Research Advisory Committee (IACUC) at Boston Children’s Hospital.

*Tcf7l2*^*Flox/Flox*^ littermates were injected retro-orbitally at five to seven weeks of age with 1 × 10^11^ genome copies of AAV8-TBG-GFP (CON, Penn Vector Core, 105535-AAV8) or AAV8-TBG-CRE (TCF7L2 L-KO, Penn Vector Core 107787-AAV8). They were then continued on a chow diet or placed on a Western Diet (Envigo, TD.88137; 42% kcal from fat, 0.2% cholesterol, and 34% sucrose by weight), or 60% High-fat Diet for twelve weeks (Research Diets Inc., D12492; 60% kcal from fat). Where noted, mice were placed in an incubation chamber at 29–31 °C.

#### 2.1.1 Glucose and lipid phenotyping

For the glucose tolerance test (GTT), animals were fasted for 14–16 h overnight, and 2 g d-glucose/kg body weight was administered intraperitoneally (*i.p.*). For the pyruvate tolerance test (PTT), mice were fasted 4 h prior to *i.p.* administration of 2 g/kg of sodium pyruvate. For the insulin tolerance test (ITT), mice were fasted 4 h prior to administration of a 1 unit/kg *i.p.* injection of human insulin. For the glutamine tolerance test (GlnTT), mice were fasted 16 h overnight before *i.p.* administration of 2 g/kg of l-Glutamine. Blood glucose levels were monitored via tail nick using a glucose meter (Bayer Contour). Values above the limit were assigned the upper limit value of 600 mg/dl. One (control group) or two (L-KO group) mice in each group became hypoglycemic during the ITT and were excluded from the analysis. Additionally, mice were food-deprived for 4 h to collect plasma samples in the fasted state using EDTA-coated Eppendorf tubes.

### 2.2 Plasma analysis

Plasma insulin was measured with an ELISA from CrystalChem (CrystalChem Ultrasensitive Mouse Insulin ELISA). Plasma triglycerides and cholesterol were measured using a colorimetric assay (Infinity Triglycerides, Infinity Cholesterol, Thermo Scientific). Plasma glutamine was measured using a colorimetric assay (Glutamine Assay Kit, Sigma).

### 2.3 Hepatic lipids

Hepatic lipids were isolated as previously described [21]. In brief, approximately 80 mg of liver was homogenized in 50 mM NaCl and extracted with 2:1 chloroform:methanol. The interphase was washed with 50 mM NaCl and 360 mM CaCl_2_ in 50% methanol. The organic extracts were mixed with 10% Triton X-100 (Sigma) in acetone, dried at room temperature overnight, and subjected to colorimetric assays (Infinity Triglycerides, Infinity Cholesterol, Thermo Scientific).

### 2.4 Bulk gene expression analysis

Total RNA was isolated from frozen liver tissue using TRIZol reagent (Life Technologies). Following isolation, RNA concentration was quantified by a NanoDrop spectrophotometer (Thermo Fisher Scientific) and reverse transcribed using the High Capacity Reverse cDNA Transcription Kit (Applied Biosystems). Reverse transcriptase quantitative polymerase chain reaction was performed using SYBR green master mix (Applied Biosystems) and 300 nM of each forward and reverse primer (obtained from MilliporeSigma, see Table 2). Fluorescence was monitored using the Applied Biosystems QuantStudio 6 flex (Thermo Fisher Scientific). Each run was followed by a melt curve (90 °C–60 °C) for quality control. Samples were analyzed in duplicate, and relative quantification of gene expression levels was performed according to the ΔΔCT method using TATA-box binding protein (*Tbp*) as reference gene. Data were expressed as 2^−ΔΔCT^ and normalized to the control group.

**Table 1:** Human phenotypic profiling of TCF7L2 rs7903146 variant within UK Biobank study participants.

rs7903146	C/C	C/T	T/T	*p*-value
n	54649	44178	9192	
Age (mean (SD))	56.6 (8)	56.6 (8)	56.6 (8)	0.835
Male (%)	25194 (46)	20420 (46)	4368 (48)	0.04
Type 2 diabetes (%)	2353 (4.3)	2508 (5.7)	698 (7.6)	<0.001
HbA1c, mmol/mol (mean (SD))	35.83 (6.3)	36.38 (7.1)	36.83 (7.6)	<0.001

Characteristics of 108,019 UK Biobank participants stratified by rs7903146 carrier status. Values are presented as either number (%) or mean (SD). Nominal *p*-values calculated as either Chi-squared test for categorical variables or ANOVA for continuous variables.

**Table 2:** RT-qPCR Primers.

Gene	Forward sequence	Reverse sequence
*Tbp*	ACCCTTCACCAATGACTCCTATG	TGACTGCAGCAAATCGCTTGG
*Tcf7l2* (Exon 1)	TTGACCAGCGAGGACTTGAC	CGCTAACGACGAGCTGATCT
*Axin2*	CAAGTGCAAACTCTCACCCA	TTGACTGGGTCGCTTCTCTT
*G6pc*	TGGCTTTTTCTTTCCTCGAA	TCGGAGACTGGTTCAACCTC
*Pepck*	TGGATGTCGGAAGAGGACTT	TGCAGGCACTTGATGAACTC
*Acly*	AAGAAGGAGGGGAAGCTGAT	TCGCATGTCTGGGTTGTTTA
*Acaca (Acc)*	TGACAGACTGATCGCAGAGAAAG	TGGAGAGCCCCACACACA
*Fasn*	GCTGCGGAAACTTCAGGAAAT	AGAGACGTGTCACTCCTGGACTT
*Cps1*	ACATGGTGACCAAGATTCCTCG	TTCCTCAAAGGTGCGACCAAT
*Otc*	GGACAGTGGAATTGCTCTCC	GTCTGTCAGCAGGGATACCAT
*Ass1*	TGCTCTAGAATGTCCAGCAAGGGCTCTG	GCGTCGACCTATTTGGCAGTGACCTTGCTC
*Asl*	CTATGACCGGCATCTGTGGAA	AGCAACCTTGTCCAACCCTTG
*Arg2*	GGGCAGCCTCTTTCCTTTCT	GCAGGCTCCACATCTCGTAA
*Glul*	TGAACAAAGGCATCAAGCAAATG	CAGTCCAGGGTCCGGGTCTT
*Gls2*	CAGAGGGACAGGAGCGTATC	TTCTTTCGGAATGCCTGAGTC

### 2.5 Metabolite profiling and mass spectrometry

#### 2.5.1 Extraction of metabolites from liver

Polar metabolites were extracted from liver tissue in methanol and chloroform. Samples were vortexed for 15 min at 4 °C and centrifuged at 13,300 rpm for 15 min at 4 °C. The supernatant containing polar metabolites was concentrated and dried at 4 °C using a CentriVap SpeedVac (Labconco). The dried extracts were then reconstituted in 1:1 acetonitrile: water.

#### 2.5.2 LC-MS

Samples were run on a Vanquish UHPLC system (Thermo Fisher) coupled to a Q-Exactive HF-X mass spectrometer utilizing a HESI probe (Thermo Fisher) in negative ion mode. A 150 × 2.1 mm iHILIC1-(P) Classic polymeric column equipped with a 2.1 × 20 mm iHILIC1-(P) Classic Guard column (both 5 μm, 200 Å, HILICON AB) was used with Buffer A (20 mM ammonium carbonate in water, with 0.1% ammonium hydroxide) and buffer B (100% acetonitrile). A linear gradient was performed at a flow rate of 0.15 ml/min as follows: 0–23 min linear gradient from 95% B to 5% B; 23–25 min hold at 5% B, to waste from 25 to 25.5 min gradient to 95% B at 0.20 ml/min, 25.5–32.5 min hold at 95% B, and finally 32.5–33 min 95% B at 0.15 ml/min. The column was held at 25 °C, the column preheater at 30 °C and the autosampler at 4 °C. MS acquisition was performed on full scan mode over a range of 70–1000 *m*/*z* and a resolution of 60,000, an AGC target of 1e5 and a maximum injection time of 20 ms with a 5 eV in-source CID. The spray voltage was set to 3 kV, the heated capillary was set at 275 °C and the probe was set at 350 °C. The sheath gas flow rate was 40, the auxiliary gas was set at 15, and the sweep gas flow rate was set to 1. Feature extraction and peak integration were performed using Tracefinder version 4.1 (Thermo Fisher). Metabolites were identified using exact mass with a 5 ppm tolerance and retention time with reference to an in-house library of chemical standards. Peaks were manually curated.

#### 2.5.3 Analysis of metabolite data

Metabolites that passed the criteria of -log10(pval) > 1.3 (i.e., *p* < 0.05) and |log2(fold change)| >1.0 were considered significantly altered. The proportion of significant metabolites in each category was calculated and a pie chart was generated in GraphPad Prism (Version 10). MetaboAnalyst 6.0 was used for pathway analysis of significantly altered metabolites. For bar graphs, data are represented as mean +/− SEM, with significance determined by a Student's t-test. The entire data set, including metabolite category assignment, is available in Suppl. Table 1.

### 2.6 Immunoblotting

For nuclear extracts, 150 mg liver tissue was dounced 24x in a glass tissue grinder set (Corning) with pestle A and B using nuclei EZ prep buffer (Sigma Aldrich, Nuc-101) with added phosphatase inhibitor (Roche). After a 5 min incubation on ice, the sample was run through a 100 μm and then a 40 μm filter prior to centrifugation (500 x G for 5 min at 4 °C) and the supernatant was removed (cytosolic fraction). The nuclear pellet was washed once in EZ prep buffer, twice with buffer ST (5 mM Tris, 73 mM NaCl, 0.5 mM CaCl_2_, and 10.5 mM MgCl_2_), and resuspended in nuclear lysis buffer (10 mM HEPES pH 7.9, 0.4 M NaCl, 1 mM sodium EDTA, 1 mM sodium EGTA, 0.5 mM DTT). Samples were agitated gently at 4 °C for 1 h and then centrifuged (13,000 rpm at 4 °C for 10 min). The supernatant was used for immunoblotting.

For whole cellular lysates, 50 mg liver tissue was homogenized in RIPA protein lysis buffer supplemented with phosSTOP and cOmplete (Roche). Samples were centrifuged (13,000 rpm at 4 °C for 30 min) and supernatant was collected for immunoblotting.

Protein quantification measurements were performed using the Pierce BCA Protein Assay Kit (Thermo Fisher). Lysates were supplemented with 5X Laemmli sample buffer (10% SDS, 312.5 mM Tris pH 6.8, 0.01% Bromophenol Blue, 50% Glycerol) with freshly added β-mercaptoethanol to 5%, and then boiled. 10–40 μg of protein was subjected to SDS-PAGE and transferred onto a PVDF membrane (Thermo Scientific). Membranes were incubated with 5% milk in 0.1% TBS-T or StartingBlockTM Blocking Buffer (Thermo Scientific) for 1 h at room temperature and then overnight at 4 °C. The following primary antibodies were used: TCF7L2 (Cell Signaling, CST2569, 1:1000), NUP98 (Cell Signaling, CST2598, 1:1000), Vinculin (Cell Signaling, CST13901, 1:1000) and Total OXPHOS Rodent WB Antibody Cocktail (Abcam, ab110413, 1:1000). The following secondary antibodies were used: anti-rabbit HRP (Cell signaling, 1:15,000) and anti-Mouse HRP (Invitrogen, 1:15,000). Finally, proteins were detected using SuperSignal West Pico PLUS or DURA Chemiluminescent Substrate (Thermo Scientific).

### 2.7 Single nucleus sequencing

#### 2.7.1 Single nucleus sequencing analysis

Nuclei were isolated as previously described [22]. 10X genomics CellRanger Count (v6.1.2) [23] was used to align FASTQs against the mouse genome (mm39) [24]. Ambient RNA was removed with CellBender (v0.2.0) [25], and gene-by-cell count matrices were generated using Seurat (v4.3.0.1) [[26], [27], [28], [29], [30]]. scDblFinder (v1.14.0) [31] was used to identify and remove doublets, and nuclei with less than 500 detected genes (nFeatures) were removed. Additionally, nuclei with at least 2% of reads mapping to mitochondrial genes (mt.percent) were discarded. The resulting 24,175 nuclei were then normalized using SCTransform [32,33], and the 3000 most variable features were used for discerning the integration anchors, and the following integration. The first twelve components of principal component analysis were used for finding nearest neighbors, and the Louvain method was used for graph-based clustering (resolution of 0.12). Known marker genes were used to assign cell types [34]. Further analysis focused on the 18,242 nuclei in the hepatocyte clusters (HEP1, HEP2).

First, we identified genes that were differentially expressed in FLOX versus L-KO in each hepatocyte cluster (HEP1 and HEP2) [35,36], filtering for expression in >10% of cells for both genotypes and FDR<0.05 (Student’s t-Test, Benjamini-Hochberg) [37]. In addition, each nucleus was scored for the expression level of pericentral and periportal marker genes [38].

Second, we identified significantly zonated genes. Nuclei in the hepatocyte clusters were re-integrated and re-clustered using the top 30 PCs and a resolution of 0.9, resulting in 19 subclusters, four of which were excluded from further analysis due to their distance from the main body of hepatocytes in the UMAP analysis. The remaining 15 clusters were subjected to diffusion map dimensionality reduction with the first 30 principal components using Destiny (v3.14.0) [39]. The diffusion pseudotime (DPT) values were rescaled and renamed as diffusion pseudodistance (DPD). A lobular location was assigned to each nucleus using previously published pericentral and periportal hepatocyte signatures [38]. Diffusion pseudodistance values were then grouped into 80 bins and a generalized additive model was created for each gene and genotype using gam (v 1.22–2) [40]. The genes were then classified as significantly zonated if *P* < 0.05 based on a parametric ANOVA of the model in either or both genotypes.

Finally, to identify gene expression modules, we performed unsupervised hierarchical clustering on all significantly zonated genes (hclust, cutree, stats v4.3.1) [41,42]. Here, diffusion pseudodistance values were grouped into 8 bins for each genotype. The resulting modules were subjected to Enrichr [41,[43], [44], [45]] analysis. Alternatively, we performed hypergeometric overlap analysis [46] with the genes known to be induced or suppressed by β-catenin [47]. For example, we determined the number of genes present in both Module A and the set of genes suppressed by β-catenin, and divided by the number of genes in both sets. *P* values were adjusted using the Benjamini Hochberg procedure [37].

### 2.8 Immunofluorescence and smFISH

#### 2.8.1 Sectioning

The right posterior lobe of the liver was fixed in 10% neutral buffered formalin and incubated at 4 °C overnight. Samples were washed four times in phosphate-buffered saline (PBS), then stored in 70% ethanol until further processing. Paraffin embedding and cutting (5 μm sections), as well as hematoxylin and eosin staining, were performed by the Beth Israel Deaconess Histology Core.

#### 2.8.2 Single molecule in situ hybridization

*In situ* hybridization was performed on 5 μm formalin fixed, paraffin embedded (FFPE) sections using RNAscope multiplex fluorescent V2 assay (323110, Advanced Cell Diagnostics, Inc). The following probes were used: Mm-Cps1-C2 (437201-C2); Mm-Gls2-C3 (449281-C3), and Mm-Glul-C1 (426231). Tissue pretreatment was performed according to the manufacturer’s instructions for FFPE murine liver tissue. Hybridization, downstream amplification steps, and DAPI staining/mounting were performed according to the manufacturer’s protocol (UM 323100). Transcripts were labeled with PerkinElmer TSA Plus fluorophores. Glul-C1 probe was detected using TSA Plus fluorescein (NEL741E001KT); Cps1-C2 probe was detected with TSA Plus Cyanine 3 (NEL744E001KT), and Gls2-C3 probe was detected with TSA Plus Cyanine 5 (NEL745E001KT).

#### 2.8.3 Image collection and downstream analyses

Images were collected at 20X with a Nikon upright Eclipse90i microscope; five Z-stacks with steps of 1 μm were combined to form the focused image, and downstream adjustments were performed in ImageJ. In all cases, images were collected using the same exposure time across samples, and downstream adjustments were performed equally across all experimental groups. One to five images were collected per mouse. Red blood cells within the lumens of the veins were masked. Quantification was done using custom Python scripts [48] (please see: 2.11 Data availability). Transcripts were detected by the Big-FISH Python package [49]. Central veins were identified by the marker gene *Glul* and/or absence of a bile duct. Central and portal veins were outlined in CellProfiler [50]. Transcripts were assigned to 10 bins based on distance from the central vein relative to the sum of distances to the central and portal veins. The counts in each bin were normalized by the area of the bin in pixels, and the percentage of total counts in each bin was calculated. Values were then averaged by mouse.

### 2.9 Human genetics studies

The UK Biobank is a large population-based biobank consisting of extensive genetic and phenotypic data for 502,629 participants [51]. Detailed baseline plasma metabolic biomarker quantification was performed in 118,461 of these patients [52,53]. 10,442 individuals were excluded based upon sample-level reliability metrics and availability of phenotypic data. Metabolite or disease associations with the rs7903146 variant in the TCF7L2 gene were tested in the 108,019 remaining individuals using linear or logistic regression adjusted for participant age at baseline assessment, sex, and the top ten principal components of genetic ancestry. Supplemental Table 5 lists the phenotypes assayed. Informed consent was obtained for all UK Biobank study participants, and analysis was approved by the Mass General Brigham Health Care Institutional Review Board (under protocol 2013P001840; UK Biobank application 7089).

### 2.10 Statistical analysis of phenotypic data and gene expression

Data are represented as mean +/− SEM, unless stated otherwise. For comparisons between two groups, a Student's t-test in GraphPad Prism (Version 10) was performed.

### 2.11 Data availability

All data are available in the main text or the supplementary materials, with the exception of the Single Nucleus Sequencing data (GEO: GSE298551). Custom R and Python scripts can be found at https://github.com/WO-Connor/TCF_Knockout/.

## 3 Results

### 3.1 Phenotyping of mice with liver specific knockout of *Tcf7l2*

Mice harboring floxed alleles of exon 1 of the *Tcf7l2* gene were injected with adeno-associated virus encoding Cre recombinase under the *Tbg* promoter to generate mice with liver specific knockout (L-KO) of *Tcf7l2*. For controls (CON), we used littermates injected with adeno-associated virus in which green fluorescent protein was substituted for the *Cre* recombinase.

**Figure 1:**
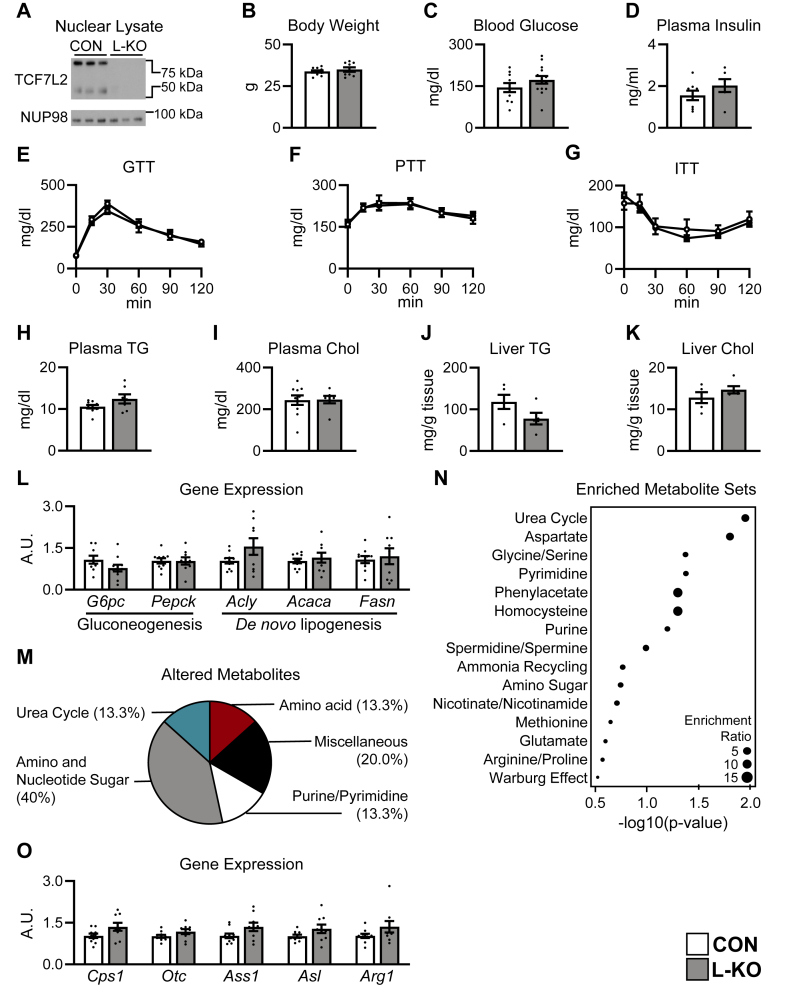
***Tcf7l2* L-KO mice show widespread metabolite changes though glucose and lipid homeostasis are largely intact.** Six-to eight-week old male *Tcf7l2*^*Flox/Flox*^ mice were injected with adeno-associated virus encoding either GFP (CON) or Cre (L-KO) and placed on a Western diet for twelve weeks. (**A**) Western blot of liver nuclear fractions. (**B**) Final body weight. (**C, D**) Four hour fasting (**C**) blood glucose and (**D**) plasma insulin. (**E-G**) Blood glucose levels during a (**E**) glucose tolerance test (GTT), (**F**) pyruvate tolerance test (PTT), and (**G**) insulin tolerance test (ITT). Four hour fasting (**H**) plasma triglycerides (TG) and (**I**) plasma cholesterol (Chol). (**J**) Liver triglycerides (TG). (**K**) Liver cholesterol (Chol). (**L, O**) Liver QPCR analysis. (**M, N**) Livers were subjected to metabolite profiling, and 30 significantly altered metabolites were identified. (**M**) Proportion of significantly altered metabolites in each category. (**N**) Metabolite Set Enrichment Analysis of significantly altered metabolites. Data are presented as the mean ± SEM; *n* = 5–14/group. A.U., arbitrary units.

In the absence of any overt phenotype on the chow diet (Supplemental Fig. 1), we subjected mice to a Western diet (34% sucrose by weight; 42% fat; 0.2% cholesterol). Figure 1A shows an immunoblot of nuclear liver extracts. Using an antibody against an epitope close to the HMG-box DNA binding domain [54], we observed two major bands at 58 and 79 kDa in the control livers, as previously reported [15]. These were undetectable in the L-KO mice. We found no overt phenotype on this diet either (Figure 1B-L, Supplemental Fig. 1P). Body weight was unchanged. Blood glucose was measured in the *ad libitum* fed state (data not shown); in the 4 h fasted state; during a glucose tolerance test; during a pyruvate tolerance test; and during an insulin tolerance test. We further stressed the mice by placing them in a thermoneutral environment, feeding them a high fat diet (60% calories from fat), and subjecting them to a fasting challenge (Figure 1C, Supplemental Fig. 1C, Supplemental Fig. 2A–F). Though hyperglycemia was occasionally noted in the L-KO mice, this was not significant and reproducible over cohorts. In parallel, the bulk levels of the gluconeogenic genes were similar to controls (Figure 1L, Supplemental Fig. 1L).

Plasma and hepatic lipid levels also remained similar to controls, though there was a trend towards reduced hepatic triglycerides (Figure 1H–K, Supplemental Fig. 1H–K). In parallel, expression of lipogenic genes, assayed on whole liver homogenates using QPCR, was similar between genotypes (Figure 1L, Supplemental Fig. 1L).

We next performed metabolomics analysis, measuring 183 metabolites in the livers of control and L-KO mice. Interestingly, principal component analysis revealed a clear separation by genotype, indicating significant metabolic derangements in the *Tcf7l2* knockout mice on both the chow and Western diet (data not shown). We then focused on the 30 metabolites that were significantly altered by genotype (Supplemental Table 1). Interestingly, more than one third of these were related to amino acid metabolism (Figure 1M,N; Supplemental Fig. 1M,N). When we performed metabolite enrichment analysis, the most significantly perturbed pathway was urea cycle, which is required for the catabolism of amino acids. Yet, QPCR on whole liver homogenates did not reveal robust changes in urea cycle gene expression, raising the question of how the transcription factor TCF7L2 alters metabolism (Figure 1O, Supplemental Fig. 1O).

### 3.2 Single nuclei sequencing of *Tcf7l2* L-KO mice

**Figure 2:**
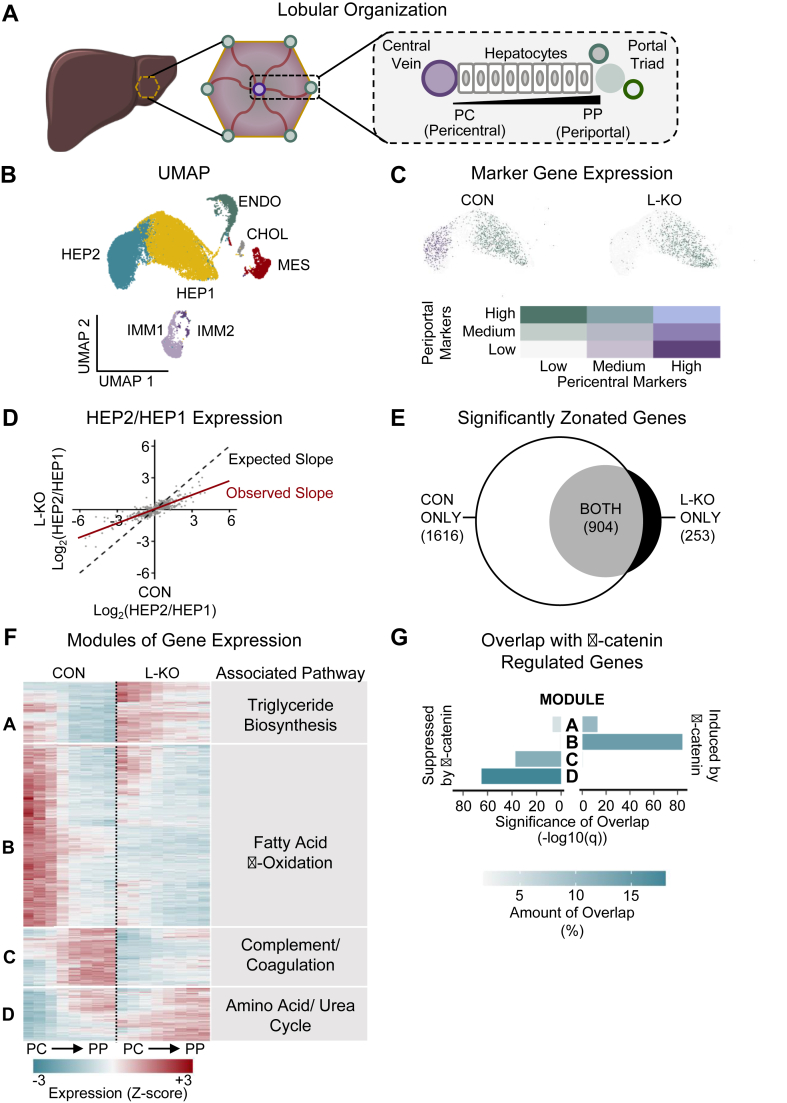
***Tcf7l2* is required for hepatic zonation.** (**A**) Diagram of the liver lobule. (**B-G**) Single nucleus sequencing was performed on the livers of five- to six-week old *Tcf7l2*^*Flox/Flox*^ mice injected with adeno-associated virus encoding either GFP (CON) or Cre (L-KO) and placed on a Western diet for twelve weeks. (**B**) UMAP projection (HEP1, hepatocyte 1; HEP2, hepatocyte 2; ENDO, endothelial cells; CHOL, cholangiocytes; MES, mesenchymal cells; IMM1, immune 1; IMM2, immune 2). (**C**) Hepatocyte clusters colored by periportal (PP) and pericentral (PC) marker gene expression [38]. (**D**) Comparison of genes that are differentially expressed in both CON and L-KO livers. (**E**) Number of significantly zonated genes by genotype. (**F**) Genes that were significantly zonated in either genotype were subjected to heatmap analysis with unsupervised clustering, generating four gene expression modules. Modules were subjected to Enrichr [43], [44], [45], [68] and a representative pathway is shown. Full Enrichr results are shown in Supplemental Table 2. (**G**) The percentage of genes in each module that are known to be suppressed or induced by β-catenin [47].

Though the liver is often considered a homogenous organ, it has a complex microarchitecture, with millions of lobules. Each lobule is anatomically defined by a central vein, surrounded by concentric layers of hepatocytes, with portal triads located at the periphery (Figure 2A). Those hepatocytes closest to the central vein (i.e., pericentral hepatocytes), and those closer to the portal vein (i.e., periportal hepatocytes) differ in their transcriptional profiles [38,55].

Since TCF7L2 has previously been suggested to have different effects in different locations in the lobule [47], we performed single nucleus sequencing on the livers of five control mice and five *Tcf7l2* L-KO mice. 29,637 nuclei were identified, of which 24,175 passed quality control. UMAP analysis was performed (Figure 2B, Supplemental Fig. 3A–3D). Both control and L-KO livers contributed similar proportions of cells to each cluster (Supplemental Fig. 3E). The clusters were presumptively identified by the presence of marker genes, with HEP1 and HEP2 as hepatocyte clusters [34]. On the one hand, in control mice, HEP1 showed high expression of periportal markers, but low expression of pericentral markers; conversely HEP2 showed high expression of pericentral markers, but low expression of periportal markers [38] (Figure 2C). These patterns identify HEP1 and HEP2 as the periportal and pericentral nuclei, respectively, and highlight the robust transcriptional differences between them. In L-KO mice, on the other hand, pericentral markers were low in both HEP1 and HEP2, and periportal markers were high in both HEP1 and HEP2. These data show a disruption in the normal zonation profiles and suggest that the transcriptional distinctions between the two hepatocyte clusters were attenuated in the knockout mice.

To determine the effects of TCF7L2 on global gene distribution, we calculated the ratio of expression of each differentially expressed gene in HEP2 versus HEP1 in each genotype. We then plotted the ratio in the knockout livers against the ratio in control livers (Figure 2D). The resulting slope was less than 1. These data suggest that the difference between HEP2 and HEP1 is diminished in the absence of TCF7L2.

To examine, with greater granularity, the effects of TCF7L2 on zonation, we performed diffusion pseudodistance analysis. Here, the distance of each nucleus from the central vein was inferred from diffusion map dimensionality reduction [38], enabling us to assess gene expression quantitatively across the lobule. This analysis showed 1616 transcripts were significantly zonated in control mice—that is, they showed a non-uniform distribution across the lobule; only 904 of these transcripts retained zonation in the knockout mice (Figure. 2E). Moreover, 253 genes became significantly zonated in knockout mice.

We next performed unsupervised clustering of the genes that were significantly zonated in either genotype. We found four gene expression modules with distinct patterns of expression across the lobule in control and L-KO mice. These modules were found by overrepresentation analysis, using Enrichr, to be associated with distinct sets of metabolic processes [41,[43], [44], [45]] (Figure 2F, Supplemental Table 2). Module A was enriched in the genes of triglyceride biosynthesis; module B was enriched in the genes of fatty acid β-oxidation; module C was enriched in the genes of complement and coagulation; and finally, module D was enriched in the genes of amino acid and urea cycle metabolism.

The activity of TCF7L2 is regulated primarily by β-catenin. β-catenin is a master transcriptional regulator that is active only in those hepatocytes which are close to the central vein. Previous studies using the livers of mice with gain and loss of β-catenin function identified 673 genes which were induced by β-catenin and 261 genes suppressed by β-catenin [47]. We determined the overlap between these β-catenin regulated gene sets and the modules identified in Figure 2F (Figure 2G, see also Supplemental Tables 3 and 4). We found that Module B was highly enriched in genes induced by β-catenin whereas Modules C and D were highly enriched in genes suppressed by β-catenin. Module A showed significant enrichment of genes that were both induced and suppressed by β-catenin [47].

### 3.3 Characterization of glutamine metabolism in *Tcf7l2* L-KO mice

**Figure 3:**
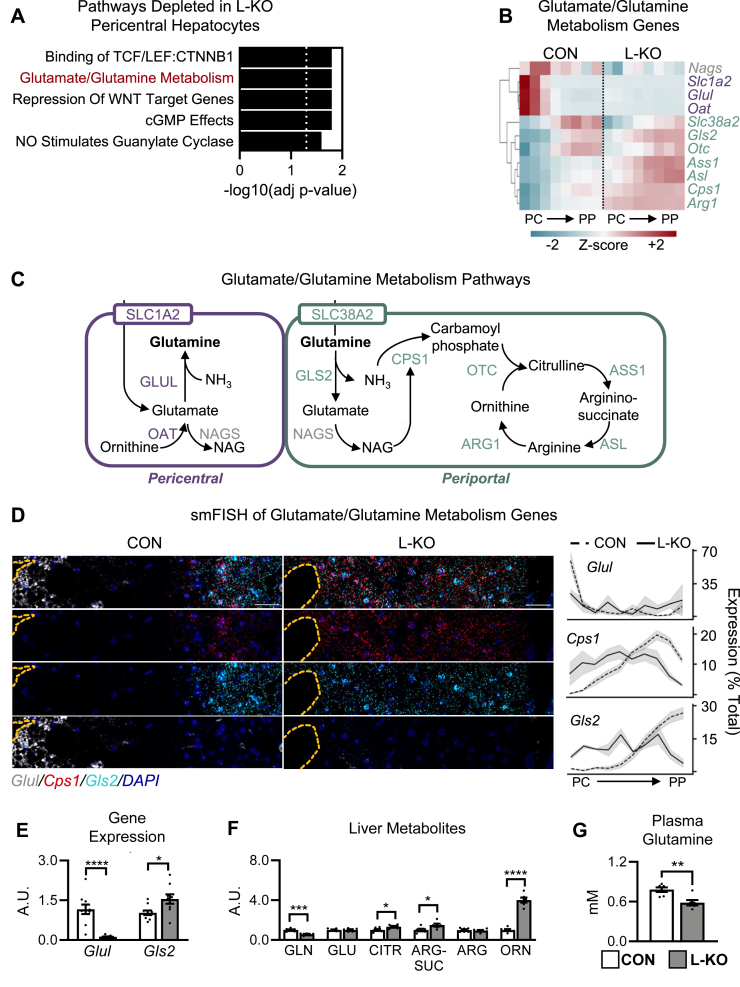
**Glutamine metabolism is perturbed in *Tcf7l2* L-KO mice.** Five- to eight-week old male *Tcf7l2*^*Flox/Flox*^ mice were injected with adeno-associated virus encoding either GFP (CON) or Cre (L-KO) and placed on a Western diet for twelve weeks. (**A**) Genes that were differentially expressed between pericentral hepatocytes (HEP2) from CON and L-KO livers by snSEQ were subjected to Enrichr analysis as described in methods. The five most significant gene sets are shown. Dotted line marks significance threshold of -log10(adj *p*-value)>1.3. (**B**) Heatmap of genes in the Glutamate/Glutamine Metabolism gene set. (**C**) Metabolic pathways. (**D**) Representative images of smFISH and quantification. (**E**) Liver QPCR analysis. (**F**) Liver metabolites. (**G**) Plasma glutamine levels. Data are presented as the mean ± SEM; *n* = 4–8/group. *P* values were determined by Student’s *t*-test; ∗*P* < 0.05, ∗∗*P* < 0.01, ∗∗∗*P* < 0.001, ∗∗∗∗*P* < 0.0001. Central vein highlighted by dashed yellow line; scale bar = 40 μm. A.U., arbitrary units. (For interpretation of the references to color in this figure legend, the reader is referred to the Web version of this article.)

To better understand the differences between L-KO and control mice we performed differential gene expression analysis within each zone [41,[43], [44], [45]], focusing on the pericentral zone (HEP2), which appeared to have the greatest changes (Figure 3A, Supplemental Fig. 4A). Among the genes in the pericentral region that were reduced by *Tcf7l2* deletion, the most overrepresented gene set was TCF/LEF signaling, as expected. The second most overrepresented gene set was glutamate/glutamine metabolism. This was particularly interesting to us given the change in amino acid and urea cycle metabolism observed by metabolomics (Figure 1M,N).

Unsupervised clustering of the genes involved in glutamate and glutamine metabolism [41,42] revealed groups of genes with distinct patterns of gene expression that, interestingly, correlated with function (Figure 3B, C). One group was enriched in the pericentral nuclei of control mice, and lost in the L-KO mice. It contained genes involved in the production of glutamine (*Slc1a2*, *Glul*, and *Oat*). Another group of genes was enriched in the periportal nuclei of control mice; in the L-KO mice, their expression was more diffuse. It contained genes involved in the consumption of glutamine (*Slc38a2*, *Gls2*, *Cps1*, *Otc*, *Ass1*, *Asl*, *Arg1*).

To confirm these changes, we performed single molecule *in situ* hybridization (smFISH). In control livers, the glutamine synthesis gene, *Glul*, was clearly expressed in the first few layers of hepatocytes surrounding the central vein; in L-KO mice, it was undetectable in any hepatocyte. In contrast, the glutamine consumption gene *Gls2*, as well as the urea cycle enzyme *Cps1*, were restricted to the periportal region in control livers but expanded in the knockout livers, mirroring the snSEQ results (Figure 3D). QPCR analysis of whole liver homogenates, which reflects the average expression across all zones, showed L-KO mice to have decreased *Glul* and increased *Gls2* (Figure 3E), -but normal levels of *Cps1* (Figure 1O).

In parallel with these gene expression changes, glutamine levels were significantly reduced in the livers of L-KO mice, whereas the urea cycle intermediates, citrulline, arginino-succinate and ornithine were significantly increased (Figure 3F). Glutamine levels were also reduced 25% in the plasma of L-KO mice (Figure 3G).

### 3.4 Phenotypic profiling of individuals with the *TCF7L2* rs7903146 variant

The profound, but unexpected, effects of *Tcf7l2* on metabolism in mice raised the question of whether TCF7L2 may also have effects beyond diabetes in humans. We therefore performed a Phenome-Wide Association Study (PheWAS), testing the association of *TCF7L2* with other metabolic phenotypes. The UK Biobank [51] contains both genetic data and baseline plasma measurements of 168 biomarkers, including amino acids, fatty acids, glycolysis metabolites, ketones, inflammatory markers, lipids and lipoproteins (Supplemental Table 5) [52]. Among the patients phenotyped for these biomarkers, we identified 54,649 individuals with the CC genotype, 44,178 with the CT genotype, and 9,192 with the TT genotype for the rs7903146 variant in *TCF7L2*, the strongest GWAS signal for type 2 diabetes [1]. These groups were not significantly different by age but showed slight male predominance (Table 1).

**Figure 4:**
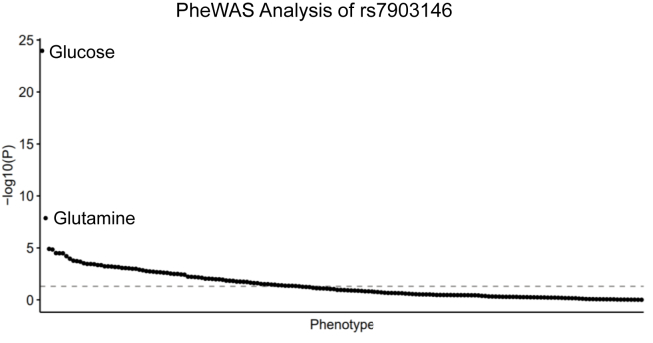
**The TCF7L2 rs7903146 variant associates with glutamine.** Human phenotypic profiling of TCF7L2 rs7903146 variant within UK Biobank study participants. Association *p*-values for rs7903146 carrier status and 168 NMR-derived biomarkers. -log10(*p*-values) were calculated using linear regression models. Dashed gray line shows Bonferroni-corrected threshold for significance (*p* = 2.97e-04).

As expected, the SNP was positively associated with type 2 diabetes (Table 1, odds ratio per T allele, 1.352 [95% CI, 1.298–1.408], *p* = 1.89e-168). The biomarker showing the strongest association was glucose, which was positively associated with the T allele (Figure 4; effect estimate per T allele, 0.055 mmol/l [0.045–0.066], *p* = 1.44e-47). The biomarker showing the second strongest association was glutamine (effect estimate per T allele, −2.137 μmol/l [−2.874–1.399], *p* = 1.37e-08), which was negatively associated with the T allele. The association with glutamine remained significant even after adjusting for diabetes status, as well as age and sex (*p* = 2.79e-06).

## 4 Discussion

Mice with liver-specific knockout of *Tcf7l2* show zone-specific disruptions in gene expression. In particular, the genes for glutamine synthesis, which are normally located pericentrally, are lost, and the genes for glutamine degradation/urea cycle, which are normally periportal, are expressed by more hepatocytes. In parallel, glutamine is decreased in the livers and plasma of knockout mice and the plasma of individuals harboring the rs7903146 variant in *TCF7L2*. Thus, these studies reveal a critical role for TCF7L2 in organizing gene expression in the liver and maintaining metabolic homeostasis.

Previous studies *in vitro* and using bulk liver homogenates have shown a variety of direct and indirect interactions of TCF7L2 with other transcriptional regulators, particularly β-catenin [10,17,18,47]. β-catenin can only enter the nucleus, interact with TCF7L2, and regulate gene expression in the pericentral hepatocytes [56]. This is due to at least two factors. First, the WNT ligands that activate β-catenin--particularly WNT2 and WNT9b, which are required for zonation [56] --are expressed in the central vein endothelial cells and have a very limited signaling radius, potentially due to their lipidation and limited diffusion capability [12,57]. Second, WNT signaling is suppressed in the periportal region due to expression of Wnt antagonists [58,59]. In addition, *Tcf7l1*, a potential repressor of TCF7L2, has been reported to be periportally enriched [[60], [61], [62]]. Consequently, TCF7L2 can only activate β-catenin targets in the pericentral hepatocytes.

Chromatin immunoprecipitation studies in the livers of mice with mutations in β-catenin have elegantly shown that TCF7L2 binds to a distinct set of promoters in the presence versus absence of β-catenin [47]. Our data are consistent with this, and suggest a model in which β-catenin, TCF7L2 and other transcription factors interact to produce at least four distinct gene expression modules. Module B represents a set of genes that are activated by the β-catenin/TCF7L2 complex; these genes are pericentrally enriched in controls, suppressed by *Tcf7l2* deletion and enriched in genes that are activated by β-catenin. Module D represents genes that are suppressed by the β-catenin/TCF7L2 complex; these genes are periportally enriched in controls, induced by *Tcf7l2* deletion, and enriched in genes that are suppressed by β-catenin. Module C represents genes that are activated by TCF7L2 via a distinct complex that is disrupted by β-catenin; these genes are periportally enriched in controls, suppressed by *Tcf7l2* deletion, and enriched in genes that are suppressed by β-catenin. Module A represents a heterogenous group that may involve two or more transcriptional complexes.

These changes in gene expression in *Tcf7l2* L-KO mice are associated with widespread perturbations in metabolism, particularly glutamine metabolism. On the one hand, expression of the glutamine degrading enzyme *Gls2* is no longer confined to the periportal hepatocytes in L-KO mice and bulk transcript levels are increased. Furthermore, the urea cycle genes, which are required for the disposal of the ammonia generated by glutamine catabolism, are ectopically expressed, and several urea cycle metabolites are increased. On the other hand, the genes for glutamine production, particularly *Glul*, normally expressed in the pericentral hepatocytes, are lost. Consequently, *Tcf7l2* L-KO mice show a 42% and 25% decrease in glutamine in their livers and plasma, respectively.

Regarding lipid and glucose metabolism, the effects of hepatic *Tcf7l2* deletion is modest. Although L-KO mice also show increased expression of the lipogenic genes and decreased expression of the fatty acid oxidation genes in the pericentral hepatocytes, plasma and hepatic lipids are unchanged. Moreover, neither bulk levels of gluconeogenic gene expression, the distribution of the gluconeogenic genes across the lobule (Supplemental Fig. 4B), nor blood glucose levels are affected by *Tcf7l2* deletion. Given the close genetic association of *TCF7L2* with diabetes, we further studied L-KO mice at multiple time points, on different obesogenic diets and after fasting. Yet, glucose levels were largely normal. We even considered the impact of deranged glutamine metabolism on glucose levels. Glutamine can potentially compensate for glucose in maintaining tricarboxylic acid cycle intermediates in the mitochondria [63] and fuel gluconeogenesis [64]. L-KO mice expressed normal levels of the mitochondrial oxidative phosphorylation complexes (Supplemental Fig. 5A), and, importantly, showed glucose levels similar to controls even during a glutamine challenge test (Supplemental Fig. 5B).

The phenotype of markedly perturbed glutamine metabolism in the absence of overt changes in glucose and lipid metabolism is also observed in females, showing that the role of hepatic TCF7L2 is not sex-specific (Supplemental Fig. 6). We would also note that during the revision of our manuscript, a different model of hepatic *Tcf7l2* disruption was published [62]. Again, marked changes in zonation and glutamine metabolism were observed, but glucose levels were normal [62].

Taken together, these studies suggest that the major effect of TCF7L2 on glucose homeostasis is mediated not by the liver but by extrahepatic tissues, potentially the β-cell. Nonetheless, it remains possible that the defects in glutamine metabolism produced by *Tcf7l2* disruption in the liver may indirectly promote metabolic dysfunction, as low glutamine levels can impair β-cell function [65,66], and glutamine supplementation in humans appears to have beneficial metabolic effects [67]. The fact that we did not observe changes in insulin levels in our studies, or changes in glucose after glutamine challenge (Supplemental Fig. 6B) suggests that these indirect effects are insufficient on their own to produce diabetes. Instead, our data suggest that in TCF7L2-associated diabetes, the loss of *Tcf7l2* in the β-cells and adipose may be the primary drivers of diabetes, with low glutamine levels due to loss of *Tcf7l2* in the hepatocytes as an aggravating factor. Consistent with this, we find that the rs7903146 variant of *TCF7L2* is associated with elevated plasma glucose as well as decreased plasma glutamine.

## 5 Conclusions

Our data indicate that TCF7L2 plays a central role in the organization of transcription within the liver lobule. While its direct effects on glucose homeostasis are modest, disruption of hepatic TCF7L2 produces widespread disruption of metabolism, particularly glutamine metabolism. Furthermore, the *TCF7L2* rs7903146 variant is associated with decreased plasma glutamine in humans. Taken together, these data indicate that TCF7L2 is a central regulator of glutamine metabolism.

## CRediT authorship contribution statement

**Joanna Krawczyk:** Investigation, Writing – review & editing. **William O’Connor:** Investigation, Methodology, Visualization, Writing – original draft, Writing – review & editing. **Pedro Vendramini:** Investigation. **Mareike Schell:** Investigation, Visualization, Writing – original draft, Writing – review & editing. **Kiran J. Biddinger:** Investigation, Methodology, Visualization, Writing – original draft, Writing – review & editing. **Matt Kanke:** Methodology. **George Pengo:** Investigation, Visualization. **Ivana Semova:** Investigation. **Tiffany Fougeray:** Investigation. **Marcia Haigis:** Methodology. **Krishna G. Aragam:** Supervision. **Wouter H. Lamers:** Writing – review & editing. **Linus T. Tsai:** Methodology. **Praveen Sethupathy:** Methodology. **Sudha B. Biddinger:** Conceptualization, Funding acquisition, Methodology, Project administration, Supervision, Visualization, Writing – original draft, Writing – review & editing.

## Relationships

There are no additional relationships to disclose.

## Patents and intellectual property

There are no patents to disclose.

## Other activities

K.A. has consulted for Sarepta Therapeutics and reports a research collaboration with Novartis. J.K. is an employee of Source Bio, Inc. M.K. is now an employee of Amgen. I.S. is now an employee of Regeneron Pharmaceuticals.

## Financial support

10.13039/100000025National Institutes of Health, T32 training grant DK007260 (JK).

Harvard Digestive Disease Center, P30 DK034854 (Pilot and Feasibility Award to SBB, MH).

American Heart Association, 862032 (KGA).

10.13039/100000025National Institutes of Health, 5R01DK125898 (SBB) and 1K08HL153937 (KGA).

Deutsche Forschungsgemeinschaft (DFG, German Research Foundation) – Projektnummer 540879521 (MS).

## Declaration of competing interest

K.A. has consulted for Sarepta Therapeutics and reports a research collaboration with Novartis. J.K. is an employee of Source Bio, Inc. M.K. is an employee of Amgen. I.S. is an employee of Regeneron Pharmaceuticals.

## Data Availability

All data are available in the main text or the supplementary materials (please see 2.11 Data Availability).

## Abbreviations

Acaca: Acetyl-CoA carboxylase
Acly: ATP citrate lyase
Arg: Arginase
ARG: Arginine
ARG-SUC: Arginino-succinate
Asl: Argininosuccinate lyase
Ass1: Argininosuccinate synthase
CHOL: Cholangiocytes
Chol: Cholesterol
CON: Control
Cps1: Carbamoyl phosphate synthetase
Cre: Cre recombinase
CTR: Citrulline
DNA: Deoxyribonucleic acid
DPD: Diffusion pseudodistance
DPT: Diffusion pseudotime
ELISA: Enzyme-linked immunosorbent assay
ENDO: Endothelial cells
Fasn: Fatty acid synthase
G6pc: Glucose-6-phosphatase
GFP: Green fluorescent protein
GLN: Glutamine
GlnTT: Glutamine Tolerance Test
Gls2: Glutaminase 2
GLU: Glutamate
Glul: Glutamine synthetase
GTT: Glucose tolerance test
HEP: Hepatocytes
IMM: Immune cells
ITT: Insulin tolerance test
LC-MS: Liquid chromatography–mass spectrometry
L-KO: Liver knockout
MES: Mesenchymal cells
Nags: N-Acetylglutamate synthase
Otc: Ornithine transcarbamylase
ORN: Ornithine
PBS: Phosphate buffered saline
PC: Pericentral
Pepck: Phosphoenolpyruvate carboxykinase
PP: Periportal
PTT: Pyruvate tolerance test
QPCR: Quantitative Real-time polymerase chain reaction
RNA: Ribonucleic acid
smFISH: Single molecule Fluorescence *in situ* hybridization
snSEQ: Single cell sequencing
SNP: Single-nucleotide polymorphism
SUC: Succinate
TG: Triglyceride

## Appendix A Supplementary data

Multimedia component 1 **Supplemental Figure 1:*****Tcf7l2*****L-KO mice on a chow diet show widespread metabolite changes though glucose and lipid homeostasis are largely intact.** Six- to eight-week old male *Tcf7l2*^*Flox/Flox*^ mice were injected with adeno-associated virus encoding either GFP (CON) or Cre (L-KO) and placed on a chow diet for twelve weeks. (**A**) Liver QPCR analysis. (**B**) Final body weight. (**C, D**) Four hour fasting (**C**) blood glucose and (**D**) plasma insulin. (**E-G**) Blood glucose levels during (**E**) glucose tolerance test (GTT), (**F**) pyruvate tolerance test (PTT), and (**G**) insulin tolerance test (ITT). Four hour fasting (**H**) plasma triglycerides (TG) and (**I**) plasma cholesterol (Chol). (**J**) Liver triglycerides (TG). (**K**) Liver cholesterol (Chol). (**L, O**) Liver QPCR analysis. (**M, N**) Livers were subjected to metabolite profiling, and 35 significantly altered metabolites were identified. (**M**) Proportion of significantly altered metabolites in each category. (**N**) Metabolite Set Enrichment Analysis of significantly altered metabolites. (**P**) AUC of GTT in Western diet-fed male mice (see Figure 1 E). Data are presented as the mean ± SEM; n=5-7/group. AUC, Area Under the Curve, A.U., arbitrary units. Multimedia component 1

Multimedia component 2 **Supplemental Figure 2:*****Tcf7l2*****L-KO mice exhibit no changes in glucose metabolism under different metabolic challenges.***Tcf7l2*^*Flox/Flox*^ mice were injected with adeno-associated virus encoding either GFP (CON) or Cre (L-KO) at six- to ten- weeks of age. (**A**) Male mice were placed on chow diet for six weeks. (**B**) Male mice were placed on Western diet for six weeks. (**C**) Male mice were placed on a Western diet for three weeks and housed at thermoneutrality. (**D**) Male mice were placed on high-fat diet for twelve weeks. (**E**) Female mice were placed on chow diet for nine weeks. (**F**) Male were placed on chow diet for four weeks and fasted. Data are presented as the mean ± SEM; n=3-9/group. GTT, Glucose Tolerance Test, AUC, Area Under the Curve, A.U., arbitrary units. *P* values were determined by Student’s *t*-test; ∗*P* < 0.05. Multimedia component 2

Multimedia component 3 **Supplemental Figure 3: snRNA sequencing quality control.** Five- to six-week old male *Tcf7l2*^*Flox/Flox*^ mice were injected with adeno-associated virus encoding either GFP (CON) or Cre (L-KO) and placed on Western diet for twelve weeks. UMAP visualization by (**A**) sample; (**B**) number of genes and genotype; and (**C**) cell cycle phase. (**D**) Heatmap of cell type markers. (**E**) Cell count by cluster as a percentage of total by genotype. Multimedia component 3

Multimedia component 4 **Supplemental Figure 4: Disruption of hepatic*****Tcf7l2*****results in changes in pericentral and periportal gene expression.** Five- to eight-week old male *Tcf7l2*^*Flox/Flox*^ mice were injected with adeno-associated virus encoding either GFP (CON) or Cre (L-KO) and placed on Western diet for twelve weeks. (**A**) Genes that were differentially expressed between pericentral (HEP2) or periportal (HEP1) hepatocytes from CON and L-KO livers by snSEQ were subjected to Enrichr analysis as described in methods. The five most significant gene sets are shown. Dotted line marks significance threshold of -log10(adj *p*-value)>1.3. (**B**) The distribution of gene expression across the lobule using snSEQ analysis. Multimedia component 4

Multimedia component 5 **Supplemental Figure 5: Mitochondrial proteins and glutamine challenge in*****Tcf7l2*****L-KO mice.** Five- to eight-week-old male *Tcf7l2*^*Flox/Flox*^ mice were injected with adeno-associated virus encoding either GFP (CON) or Cre (L-KO) and (**A**) placed on Western diet for twelve weeks or (**B**) on chow diet for two weeks. (**A**) Western blot of mitochondrial respiration complexes (left) and quantification (right). (**B**) Glutamine Tolerance Test (GlnTT). Data are presented as the mean ± SEM; n=6-7/group. AUC, Area Under the Curve, A.U., arbitrary units. Multimedia component 5

Multimedia component 6 **Supplemental Figure 6: Disruption of hepatic*****Tcf7l2*****leads to metabolic changes in Western****diet-fed****female mice.** Six- to eight-week-old female *Tcf7l2*^*Flox/Flox*^ mice were injected with adeno-associated virus encoding either GFP (CON) or Cre (L-KO) and placed on Western diet for twelve weeks. (**A**) Liver QPCR analysis. (**B**) Final body weight. (**C, D**) Four hours fasting (**C**) blood glucose and (**D**) plasma insulin. (**E-G**) Blood glucose levels during (**E**) glucose tolerance test (GTT) and (**F**) pyruvate tolerance test (PTT). Four hour fasting (**G**) plasma triglycerides (TG) and (**H**) plasma cholesterol (Chol). (**I**) Liver triglycerides (TG). (**J**) Liver Cholesterol (Chol). (**K, M**) Liver QPCR analysis. (**L**) Representative images of smFISH and quantification. Data are presented as the mean ± SEM; n=4-8/group. *P* values were determined by Student’s *t*-test; ∗*P* < 0.05, ∗∗∗∗*P* < 0.0001. Central vein highlighted by dashed yellow line; scale bar = 40 μm. A.U., arbitrary units. Multimedia component 6
