# Supplementary material for: The diabetes gene Tcf7l2 organizes gene expression in the liver and regulates amino acid metabolism
Source: Mol Metab. 2025 Jul 15;99:102208. doi: 10.1016/j.molmet.2025.102208 (PMC12318266; doi:10.1016/j.molmet.2025.102208)
Supplement: Multimedia component 1 — Supplemental Figure 1:Tcf7l2L-KO mice on a chow diet show widespread metabolite changes though glucose and lipid homeostasis are largely intact. Six- to eight-week old male Tcf7l2Flox/Flox mice were injected with adeno-associated virus encoding either GFP (CON) or Cre (L-KO) and placed on a chow diet for twelve weeks. (A) Liver QPCR analysis. (B) Final body weight. (C, D) Four hour fasting (C) blood glucose and (D) plasma insulin. (E-G) Blood glucose levels during (E) glucose tolerance test (GTT), (F) pyruvate tolerance test (PTT), and (G) insulin tolerance test (ITT). Four hour fasting (H) plasma triglycerides (TG) and (I) plasma cholesterol (Chol). (J) Liver triglycerides (TG). (K) Liver cholesterol (Chol). (L, O) Liver QPCR analysis. (M, N) Livers were subjected to metabolite profiling, and 35 significantly altered metabolites were identified. (M) Proportion of significantly altered metabolites in each category. (N) Metabolite Set Enrichment Analysis of significantly altered metabolites. (P) AUC of GTT in Western diet-fed male mice (see Figure 1 E). Data are presented as the mean ± SEM; n=5-7/group. AUC, Area Under the Curve, A.U., arbitrary units. [file mmc1.pptx]

## Slide 1
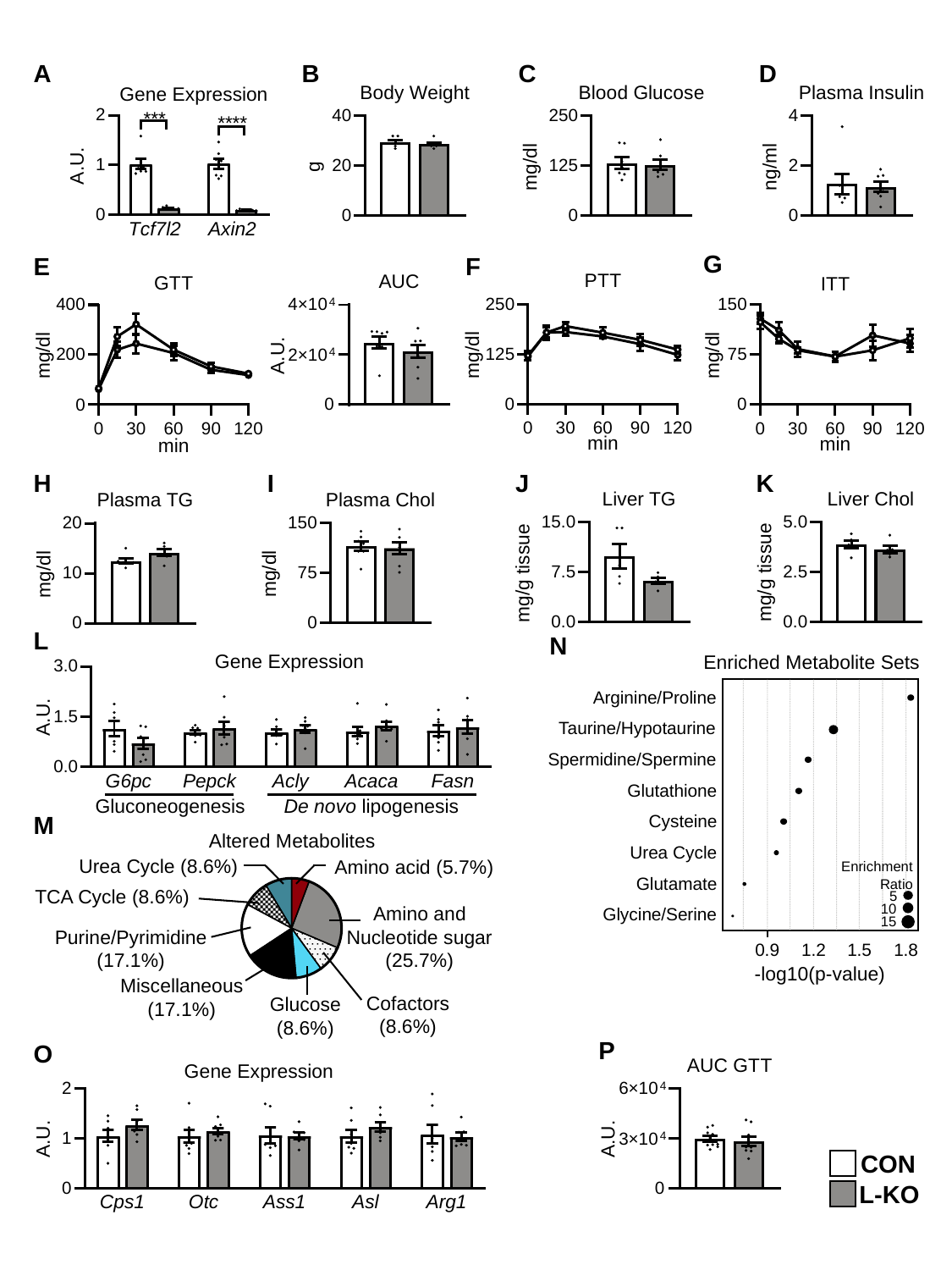

A
B
C
D
G
E
F
H
I
J
K
L
N
Enriched Metabolite Sets
Arginine/Proline
Taurine/Hypotaurine
Spermidine/Spermine
Glutathione
Cysteine
Urea Cycle
Enrichment
Ratio
5
10
15
Glutamate
Glycine/Serine
0.9
1.2
1.5
1.8
-log10(p-value)
Gene Expression
Gluconeogenesis
De novo lipogenesis
M
Altered Metabolites
Urea Cycle (8.6%)
Amino acid (5.7%)
TCA Cycle (8.6%)
Amino and
Nucleotide sugar
(25.7%)
Purine/Pyrimidine
(17.1%)
Miscellaneous
(17.1%)
Cofactors
(8.6%)
Glucose
(8.6%)
P
O
Gene Expression
CON
L-KO
