# Supplementary material for: The diabetes gene Tcf7l2 organizes gene expression in the liver and regulates amino acid metabolism
Source: Mol Metab. 2025 Jul 15;99:102208. doi: 10.1016/j.molmet.2025.102208 (PMC12318266; doi:10.1016/j.molmet.2025.102208)
Supplement: Multimedia component 2 — Supplemental Figure 2:Tcf7l2L-KO mice exhibit no changes in glucose metabolism under different metabolic challenges.Tcf7l2Flox/Flox mice were injected with adeno-associated virus encoding either GFP (CON) or Cre (L-KO) at six- to ten- weeks of age. (A) Male mice were placed on chow diet for six weeks. (B) Male mice were placed on Western diet for six weeks. (C) Male mice were placed on a Western diet for three weeks and housed at thermoneutrality. (D) Male mice were placed on high-fat diet for twelve weeks. (E) Female mice were placed on chow diet for nine weeks. (F) Male were placed on chow diet for four weeks and fasted. Data are presented as the mean ± SEM; n=3-9/group. GTT, Glucose Tolerance Test, AUC, Area Under the Curve, A.U., arbitrary units. P values were determined by Student’s t-test; ∗P < 0.05. [file mmc2.pptx]

## Slide 1
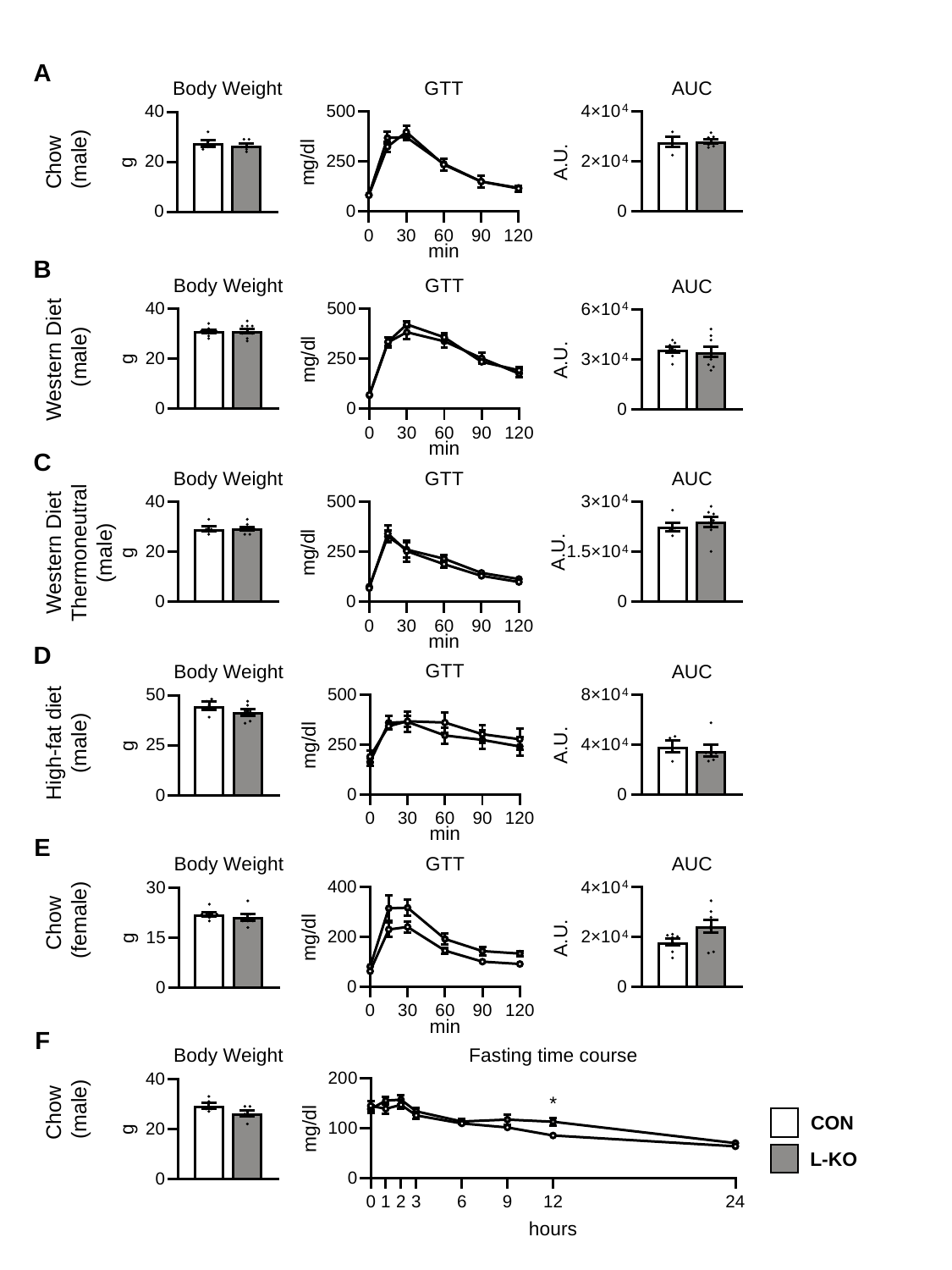

A
Chow
 (male)
Western Diet
(male)
Western Diet
Thermoneutral
(male)
High-fat diet
(male)
Chow
(female)
Chow
 (male)
B
C
D
E
F
CON
L-KO
