# Supplementary material for: The diabetes gene Tcf7l2 organizes gene expression in the liver and regulates amino acid metabolism
Source: Mol Metab. 2025 Jul 15;99:102208. doi: 10.1016/j.molmet.2025.102208 (PMC12318266; doi:10.1016/j.molmet.2025.102208)
Supplement: Multimedia component 3 — Supplemental Figure 3: snRNA sequencing quality control. Five- to six-week old male Tcf7l2Flox/Flox mice were injected with adeno-associated virus encoding either GFP (CON) or Cre (L-KO) and placed on Western diet for twelve weeks. UMAP visualization by (A) sample; (B) number of genes and genotype; and (C) cell cycle phase. (D) Heatmap of cell type markers. (E) Cell count by cluster as a percentage of total by genotype. [file mmc3.pptx]

## Slide 1
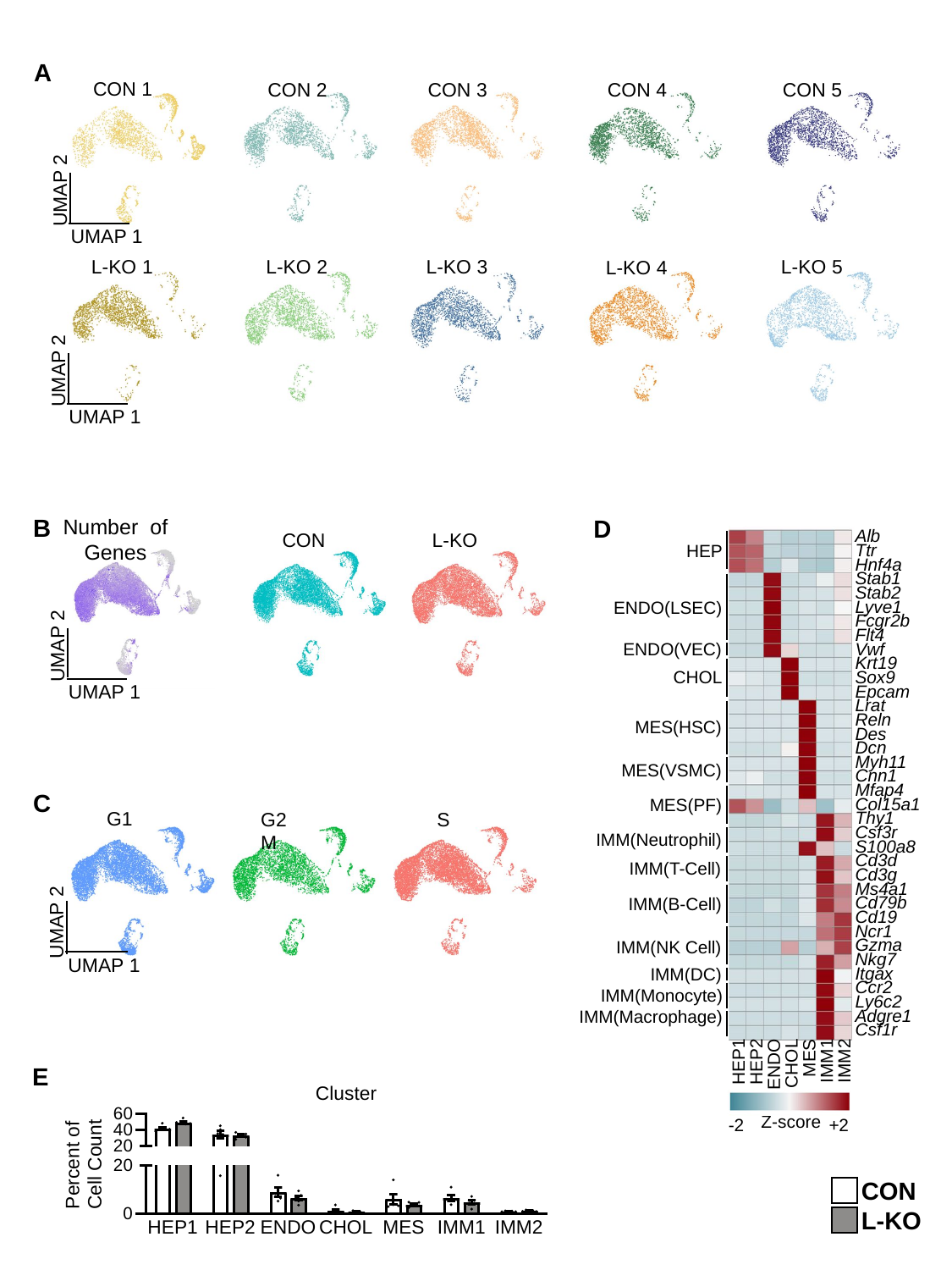

A
CON 1
CON 3
CON 5
CON 2
CON 4
UMAP 2
UMAP 1
L-KO 1
L-KO 3
L-KO 5
L-KO 2
L-KO 4
UMAP 2
UMAP 1
B
D
Alb
Ttr
Hnf4a
Stab1
Stab2
Lyve1
Fcgr2b
Flt4
Vwf
Krt19
Sox9
Epcam
Lrat
Reln
Des
Dcn
Myh11
Cnn1
Mfap4
Col15a1
Thy1
Csf3r
S100a8
Cd3d
Cd3g
Ms4a1
Cd79b
Cd19
Ncr1
Gzma
Nkg7
Itgax
Ccr2
Ly6c2
Adgre1
Csf1r
HEP
ENDO(LSEC)
ENDO(VEC)
CHOL
MES(HSC)
MES(VSMC)
MES(PF)
IMM(Neutrophil)
IMM(T-Cell)
IMM(B-Cell)
IMM(NK Cell)
IMM(DC)
IMM(Monocyte)
IMM(Macrophage)
HEP1
HEP2
ENDO
CHOL
MES
IMM1
IMM2
+2
-2
Z-score
Number of
Genes
CON
L-KO
A
UMAP 2
UMAP 1
C
G1
G2M
S
UMAP 2
UMAP 1
E
CON
L-KO
