# Supplementary material for: The diabetes gene Tcf7l2 organizes gene expression in the liver and regulates amino acid metabolism
Source: Mol Metab. 2025 Jul 15;99:102208. doi: 10.1016/j.molmet.2025.102208 (PMC12318266; doi:10.1016/j.molmet.2025.102208)
Supplement: Multimedia component 4 — Supplemental Figure 4: Disruption of hepaticTcf7l2results in changes in pericentral and periportal gene expression. Five- to eight-week old male Tcf7l2Flox/Flox mice were injected with adeno-associated virus encoding either GFP (CON) or Cre (L-KO) and placed on Western diet for twelve weeks. (A) Genes that were differentially expressed between pericentral (HEP2) or periportal (HEP1) hepatocytes from CON and L-KO livers by snSEQ were subjected to Enrichr analysis as described in methods. The five most significant gene sets are shown. Dotted line marks significance threshold of -log10(adj p-value)>1.3. (B) The distribution of gene expression across the lobule using snSEQ analysis. [file mmc4.pptx]

## Slide 1
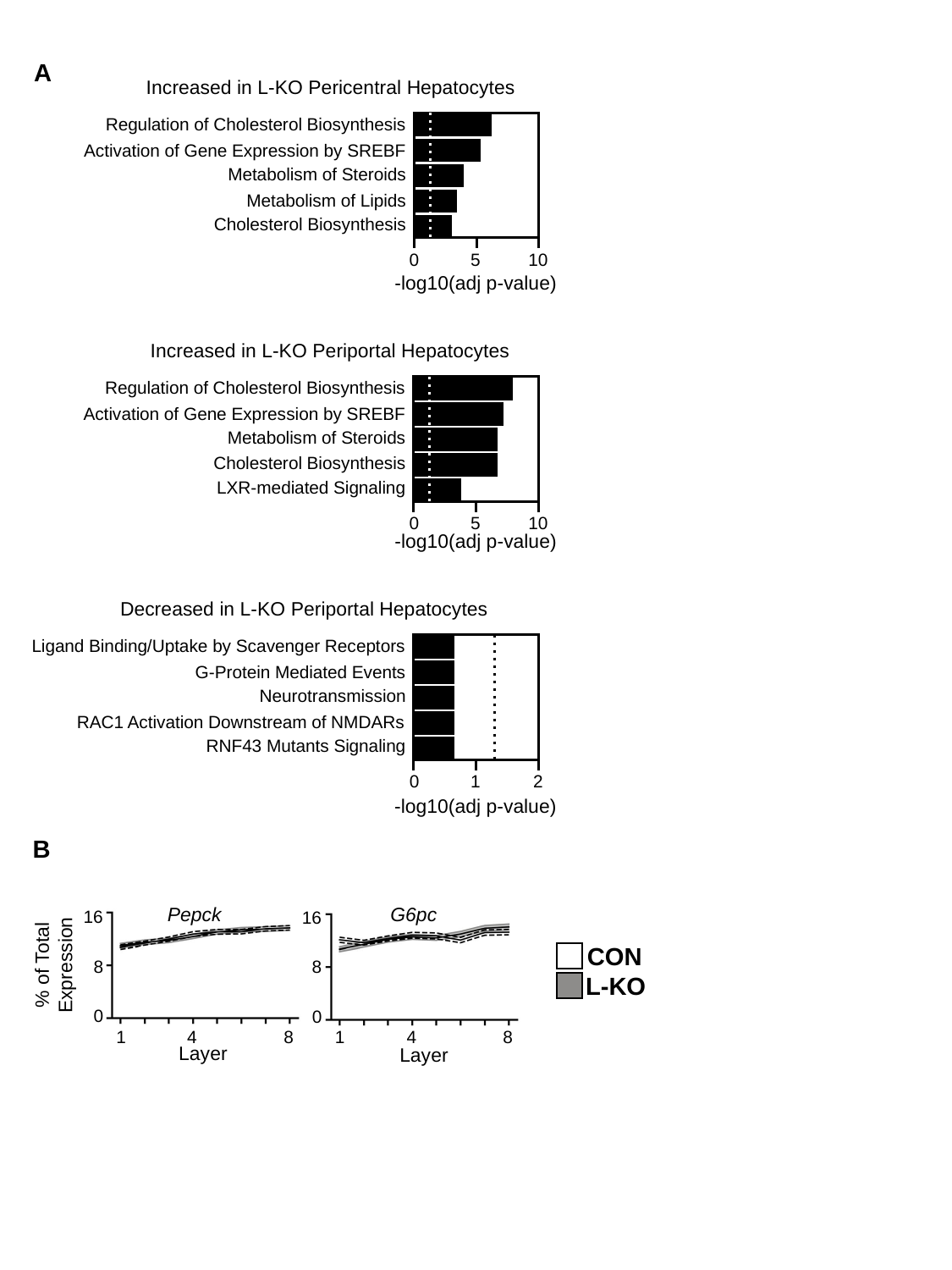

A
Increased in L-KO Pericentral Hepatocytes
Regulation of Cholesterol Biosynthesis
Activation of Gene Expression by SREBF
Metabolism of Steroids
Metabolism of Lipids
Cholesterol Biosynthesis
-log10(adj p-value)
0
5
10
Increased in L-KO Periportal Hepatocytes
Regulation of Cholesterol Biosynthesis
Activation of Gene Expression by SREBF
Metabolism of Steroids
Cholesterol Biosynthesis
LXR-mediated Signaling
-log10(adj p-value)
0
5
10
Decreased in L-KO Periportal Hepatocytes
Ligand Binding/Uptake by Scavenger Receptors
G-Protein Mediated Events
Neurotransmission
RAC1 Activation Downstream of NMDARs
RNF43 Mutants Signaling
-log10(adj p-value)
0
1
2
B
G6pc
Pepck
16
16
CON
% of Total
Expression
8
8
L-KO
0
0
1
4
8
1
4
8
Layer
Layer
