# Supplementary figures and images for: The diabetes gene Tcf7l2 organizes gene expression in the liver and regulates amino acid metabolism
Source: Mol Metab. 2025 Jul 15;99:102208. doi: 10.1016/j.molmet.2025.102208 (PMC12318266; doi:10.1016/j.molmet.2025.102208)

## Slide 1
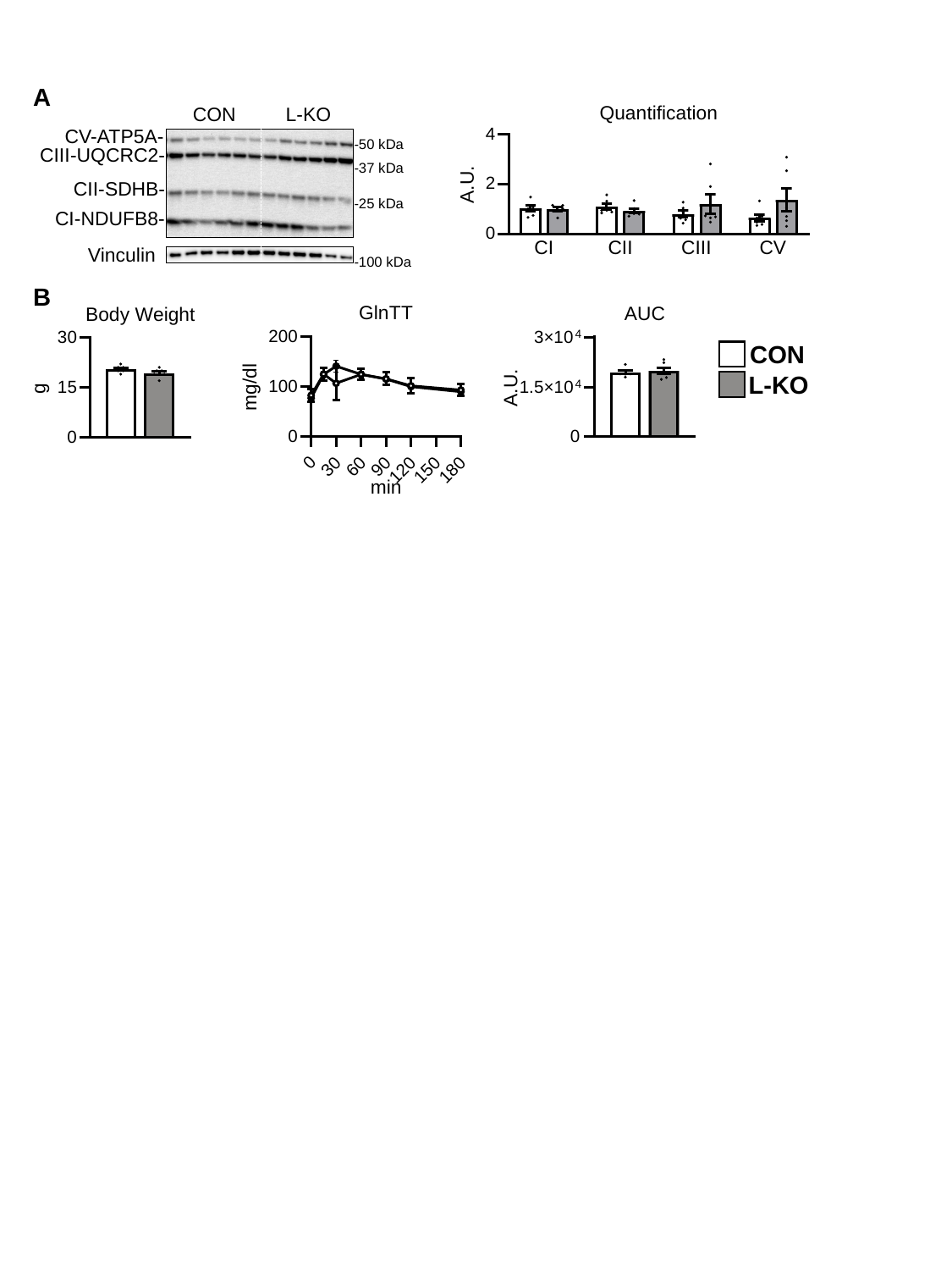

A
CON
L-KO
CV-ATP5A-
-50 kDa
CIII-UQCRC2-
-37 kDa
CII-SDHB-
-25 kDa
CI-NDUFB8-
Vinculin
-100 kDa
B
CON
L-KO

Supplement: Multimedia component 5 — Supplemental Figure 5: Mitochondrial proteins and glutamine challenge inTcf7l2L-KO mice. Five- to eight-week-old male Tcf7l2Flox/Flox mice were injected with adeno-associated virus encoding either GFP (CON) or Cre (L-KO) and (A) placed on Western diet for twelve weeks or (B) on chow diet for two weeks. (A) Western blot of mitochondrial respiration complexes (left) and quantification (right). (B) Glutamine Tolerance Test (GlnTT). Data are presented as the mean ± SEM; n=6-7/group. AUC, Area Under the Curve, A.U., arbitrary units. [file mmc5.pptx]
