# Supplementary material for: The diabetes gene Tcf7l2 organizes gene expression in the liver and regulates amino acid metabolism
Source: Mol Metab. 2025 Jul 15;99:102208. doi: 10.1016/j.molmet.2025.102208 (PMC12318266; doi:10.1016/j.molmet.2025.102208)
Supplement: Multimedia component 6 — Supplemental Figure 6: Disruption of hepaticTcf7l2leads to metabolic changes in Westerndiet-fedfemale mice. Six- to eight-week-old female Tcf7l2Flox/Flox mice were injected with adeno-associated virus encoding either GFP (CON) or Cre (L-KO) and placed on Western diet for twelve weeks. (A) Liver QPCR analysis. (B) Final body weight. (C, D) Four hours fasting (C) blood glucose and (D) plasma insulin. (E-G) Blood glucose levels during (E) glucose tolerance test (GTT) and (F) pyruvate tolerance test (PTT). Four hour fasting (G) plasma triglycerides (TG) and (H) plasma cholesterol (Chol). (I) Liver triglycerides (TG). (J) Liver Cholesterol (Chol). (K, M) Liver QPCR analysis. (L) Representative images of smFISH and quantification. Data are presented as the mean ± SEM; n=4-8/group. P values were determined by Student’s t-test; ∗P < 0.05, ∗∗∗∗P < 0.0001. Central vein highlighted by dashed yellow line; scale bar = 40 μm. A.U., arbitrary units. [file mmc6.pptx]

## Slide 1
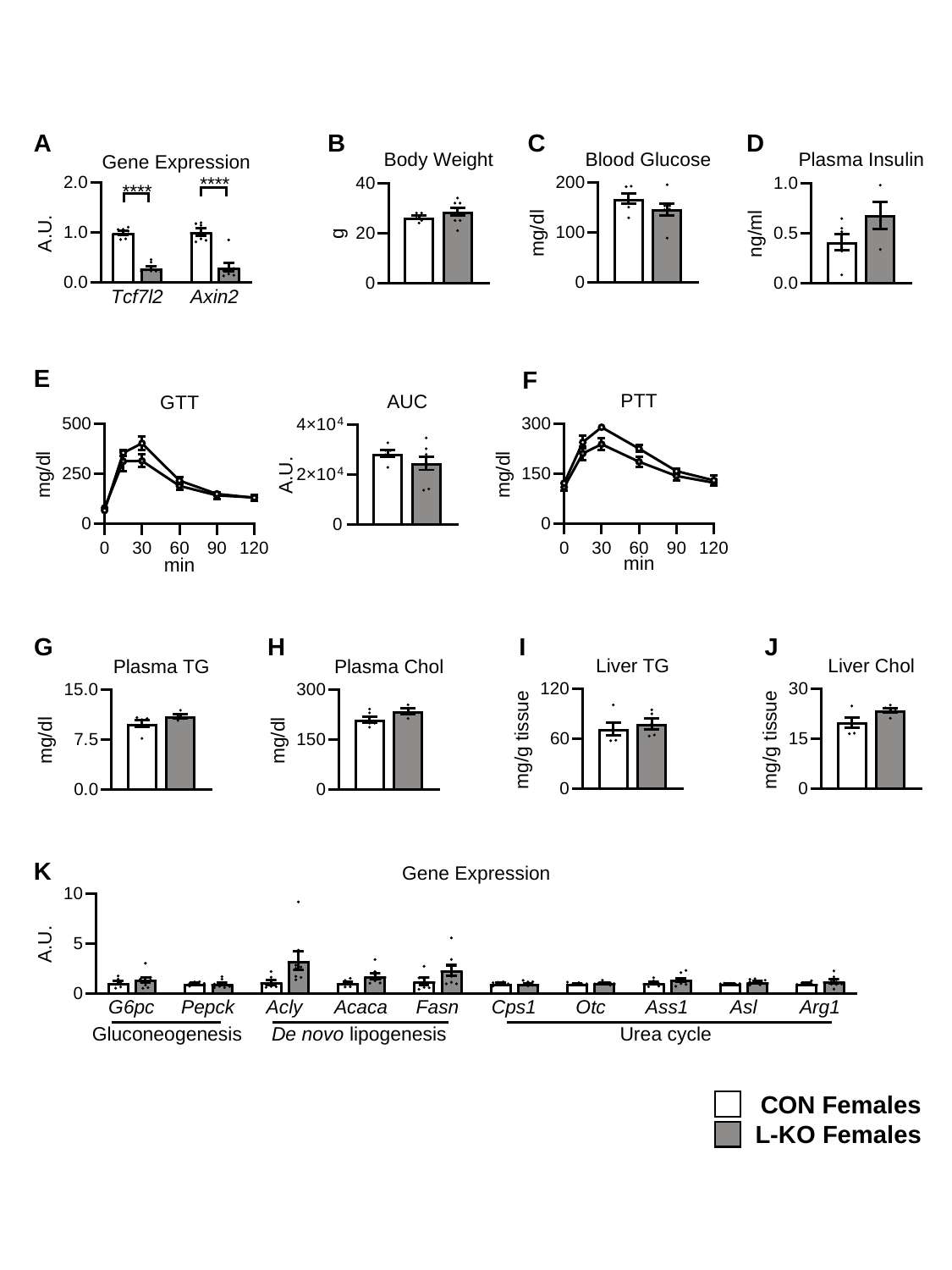

A
B
C
D
E
F
G
H
I
J
K
Gluconeogenesis
De novo lipogenesis
Urea cycle
CON Females
L-KO Females

## Slide 2
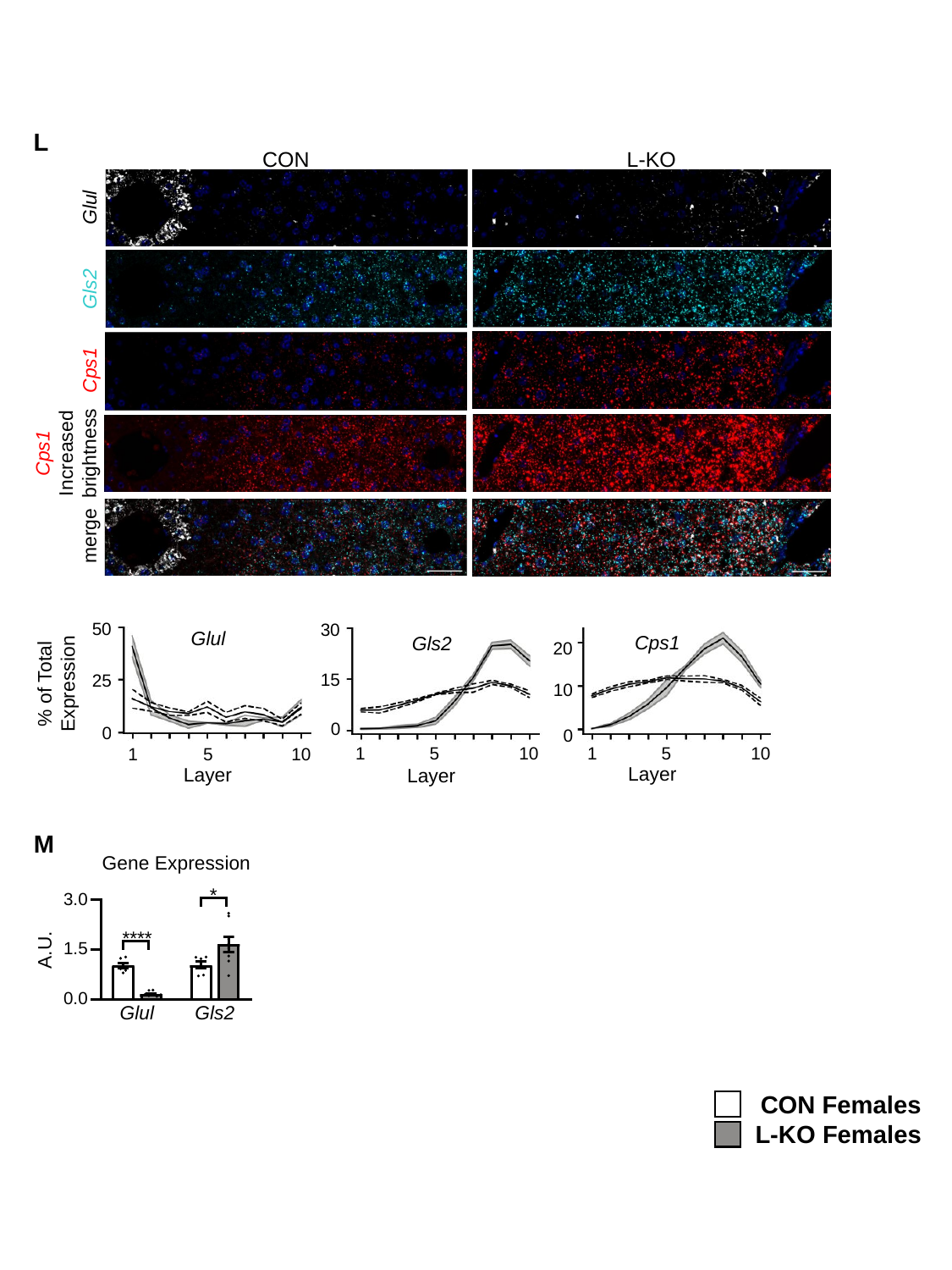

L
CON
L-KO
Glul
Gls2
Cps1
Cps1
Increased
brightness
merge
50
30
Glul
Glul
Cps1
Gls2
20
% of Total
Expression
15
25
10
0
0
0
1
5
10
1
5
10
1
5
10
Layer
Layer
Layer
M
CON Females
L-KO Females
